# Comparison of outcomes for general and local anesthesia in the management of nasal bone fractures: a meta-analysis

**DOI:** 10.1186/s40001-024-01896-3

**Published:** 2024-06-02

**Authors:** Ting Xu, Xinsheng Yi, Shitong Xia, Sihai Wu

**Affiliations:** 1 Department of Otolaryngology Head and Neck Surgery, Wuxi Clinical College, Nantong University, No. 68 Zhongshan Road, Liangxi District, Wuxi, 214002 Jiangsu China; 2 Department of Otolaryngology Head and Neck Surgery, The Second People‘s Hospital of Wuxi, Wuxi, China

**Keywords:** Nasal bone fracture, General anesthesia, Local anesthesia, Surgery, Meta-analysis

## Abstract

**Background:**

This meta-analysis aimed to perform a head-to-head comparison of the role of general anesthesia (GA) and local anesthesia (LA) in the management of patients with nasal bone fractures (NBFs).

**Methods:**

PubMed, Embase, and Web of Science were comprehensively searched. Studies investigating the clinical outcomes of GA and LA in the management of NBFs were included. Pooled odds ratios (OR) with the respective 95% confidence intervals (CIs) were calculated. Heterogeneity between the included studies was evaluated. The risk of bias in the included studies was assessed.

**Results:**

Eight studies were included in this meta-analysis. The pooled ORs for cosmetic results, residual septal deformity, the need for further surgery, patients’ satisfaction with the anesthesia procedure, and patients’ satisfaction with the surgery results were 0.70 (95% CI 0.18, 2.64; *z* = − 0.53, *p* = 0.5957), 1.11 (95% CI 0.37, 3.30; *z* = 0.18, *p* = 0.8558), 1.19 (95% CI 0.65, 2.20; *z* = 0.56, *p* = 0.5760), 1.57 (95% CI 0.92, 2.69; *z* = 1.65, *p* = 0.0982), and 1.00 (95% CI 0.55, 1.80; *z* = − 0.00, *p* = 0.9974).

**Conclusions:**

Insignificant difference on clinical outcomes was observed between GA and LA in the manipulation of patients with NBFs, and the choice of anesthetic approach should be based on the tolerability of the methods and the severity of nasal fractures.

**Supplementary Information:**

The online version contains supplementary material available at 10.1186/s40001-024-01896-3.

## Background

The nose is highly susceptible to injury because of its inherent structure and location on the human face, and nasal bone fractures (NBFs) account for over half of all facial fractures [1, 2]. The incidence of nasal fractures is higher than that of zygomatic/maxillary, orbital, and mandibular fractures [3]. Common causes of NBFs include assaults, motor vehicle accidents, falls, and sport accidents [4]. Nasal fractures are described as open or closed, provided there are exposed bones, displaced or not displaced, simple or comminuted [3]. NBFs are usually determined clinically by assessing the mechanism, location, and timing of the injury, history of nasal injury or surgery [4, 5].

The treatment of nasal fractures depends on their severity, with the main goal of using the least invasive method to restore preorbital morphology and function [5]. Patients with non-displaced fractures of the nasal bone, nasal septum, and/or anterior nasal spine, and without clinical nasal deformity or nasal obstruction, were closely observed [3, 5, 6]. When patients experience septal hematoma, reduced nasal airflow due to anatomical trauma, and/or unacceptable cosmetic conditions, surgery is required for nasal fractures [7]. Most cases of nasal fractures that require repositioning of the os nasale are first treated with closed techniques, while open reduction serves for patients with residual nasal deformities, severe fractures, and displaced-fractures [8]. Furthermore, NBFs are treated under local anesthesia (LA) or general anesthesia (GA), based on various considerations, such as the duration from trauma to evaluation and the patient’s age [9]. In the treatment of nasal fractures, GA is usually superior to local anesthesia when there is evidence to support significantly improved nasal appearance and function, reduced subsequent surgeries, and patients’ subjective satisfaction with anesthesia [10]. However, LA is associated with the following advantages: short hospitalization time, the patient's ability to see the cosmetic appearance after surgery and allowance for further optimization, minimum delay from diagnosis until treatment, lower costs, and if LA surgery is not successful, the option of GA surgery would be maintained [11].

To our knowledge, several studies have compared the roles of GA and LA in the management of NBFs [11–14]. However, the results of these studies have varied. This meta-analysis aimed to compare the effects of GA and LA in the manipulation of NBFs and to provide evidence-based clues for clinical practice.

## Methods

The meta-analysis was performed according to the Preferred Reporting Items for Systematic Reviews and Meta-analysis (PRISMA) rather than a self-designed protocol [15]. Ethical approval was declared for the original articles, and no ethical approval was required for this meta-analysis.

### Search strategy and selection criteria

Two independent authors comprehensively searched electronic databases including PubMed, Embase, and Web of Science from inception to April 30, 2023. Only articles published in English were considered. The following keywords were used for database research: nasal bone fracture, NBFs, nose bone fractures, nasal fractures, general anesthesia, and local anesthesia. The references in the reviews were manually screened for further studies. Search strategies for each online database are listed in Table S1. The inclusion criteria were as follows: (1) patients: study population included patients with NBFs; (2) intervention and comparison: both GA and LA were utilized in the same study; (3) outcomes: effect sizes with regard to cosmetic results,patients’ satisfaction with the anesthesia procedure, patients’ satisfaction with the surgery results, residual septal deformity, need for further surgery, change in airway obstruction post-manipulation, patients’ preference for treatment when refracted were reported in the GA and LA groups, or the above data can be calculated according to the results provided in the included studies; (4) study design: controlled studies. Exclusion criteria included: (1) data that could not be extracted or calculated; (2) case reports, reviews, conference abstracts, and animal studies; and (3) duplicates or overlaps in the research participants. Two independent researchers performed the literature search and study selection. Disagreements were resolved by discussion.

### Data extraction and quality assessments

Two independent reviewers screened the titles and abstracts of the citations in the first round of study selection. A full-text assessment of the studies was then conducted for the final inclusion. Moreover, in addition to the relevant outcomes mentioned above, we extracted the following parameters: first author’s name, year of publication, study design, number of participants in the GA group, number of participants in the LA group, age of participants in the GA group, and age of participants in the LA group. The Cochrane Collaboration’s tool for assessing the risk of bias was used to judge the risk of bias for randomized controlled trials (RCTs) and the Newcastle–Ottawa scale (NOS) criteria was utilized for non-randomized controlled studies [16, 17].

### Statistical analysis

We used R language and environment for statistical computing (version 4.2.1) for data analyses in the study. We calculated pooled estimates of odds ratios (ORs) and their respective 95% confidence intervals (CIs) as the effect sizes to compare the clinical outcomes of GA and LA. An OR value > 1 represents the superior effect of GA over LA; an OR value < 1 indicates the inferior effect of GA over LA; an OR value = 1 indicates equal effect of both GA and LA. I^2^ statistic was used to assess heterogeneity between the included studies. Insignificant, low, moderate, and high heterogeneity were rated by I^2^ values of 0 -, 25% -, 50% -, and 75–100%, respectively [18]. We created funnel plots and Deeks’ test for asymmetry of funnel plots to assess potential publication bias. In addition, a sensitivity analysis was performed to evaluate the impact of a single study on overall outcomes. Statistical significance was set at *p* value < 0.05.

## Results

### Study selection and characteristics

**Fig. 1:**
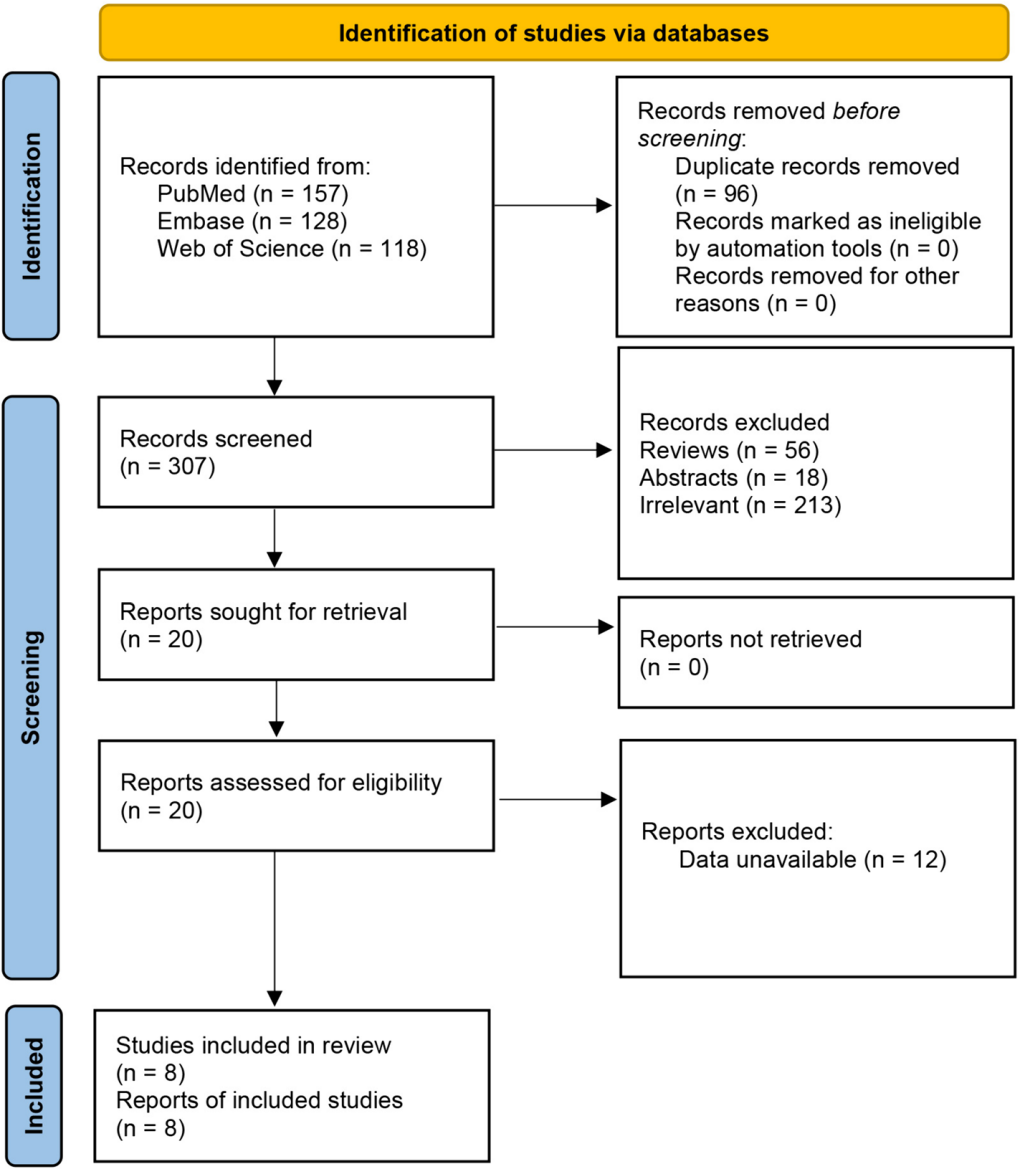
Flowchart of the literature search

A total of 403 articles were identified from the database search. After an initial screening of these citations, 96 duplicates were removed and irrelevant 287 studies were eliminated. After the full-text assessment for eligibility of the remaining 20 citations, eight articles were identified for inclusion in this meta-analysis [11–14, 19–22]. No additional studies were identified by scanning the bibliographies of the related reviews (Fig. 1). Four studies were RCTs, 2 were cohort studies, and 2 were retrospective studies (Table 1). Overall, the quality of the included studies was moderate (Tables 2 and 3). Only Cook’s study reported outcomes of airway obstruction; therefore, this outcome cannot be synthesized, and the results showed significant improvement in both the LA and GA groups after manipulation, although there was no difference between the two groups.

**Table 1:** Study characteristics

First author’s name	Year of publication	Study design	Number of participants in GA group	Number of participants in LA group	Age of participants in GA group (in year)	Age of participants in LA group (in year)
Watson	1988	RCT	12	17	22 (16–47)	24 (18–85)
Waldron	1989	Cohort study	50	50	NR	NR
Cook	1990	RCT	25	25	31.3 (18–59)	28.3 (16–65)
Ridder	2002	Retrospective study	28	68	NR	NR
Courtney	2003	Retrospective study	65	59	NR	NR
Khwaja	2007	RCT	65	74	25 (16–62)	28 (16–69)
Atighechi	2009	RCT	72	68	NR	NR
Kyung	2018	Cohort study	27	17	31.1	22.1

*RCT* randomized controlled trial, *NR* not reported

**Table 2:** Cochrane criteria for quality of RCT

Study	Random sequence generation (selection bias)	Allocation concealment (selection bias)	Blinding of participants and personnel (performance bias)	Blinding of outcome assessment (detection bias)	Incomplete outcome data (attrition bias)	Selective reporting (reporting bias)	Other bias
Watson et al. [12]	Unclear	Unclear	High	High	Low	Low	Unclear
Cook et al. [11]	Low	Unclear	High	Unclear	Low	Low	Unclear
Khwaja et al. [14]	Unclear	Unclear	High	Unclear	Low	Low	Unclear
Atighechi et al. [20]	Low	Unclear	High	Low	Low	Low	Unclear

**Table 3:** NOS criteria for quality of non-randomized controlled studies

Study	Representativeness of the exposed cohort	Selection of the non-exposed cohort	Ascertainment of exposure	Demonstration that the outcome of interest was not present at the start of the study	Comparability of cohorts on the basis of the design or analysis	Assessment of outcome	Was follow-up long enough for outcomes to occur	Adequacy of follow-up of cohorts	Total quality scores
Waldron et al. [22]	*	*		*	**	*	*	*	8
Ridder et al. [21]	*	*		*	**	*	*	*	8
Courtney et al. [13]	*	*		*	**	*	*	*	8
Kyung et al. [19]	*	*		*	**	*	*	*	8

Stars stand for the score of NOS, the maximum score on the NOS is 9 (highest quality), and we assigned scores of 0–3, 4–6, and 7–9 for low, moderate, and high quality of studies, respectively

### Pooled analyses

**Fig. 2:**
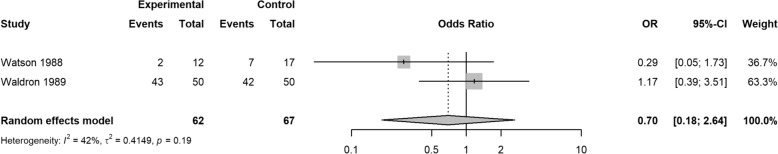
Forest plot of cosmetic results

**Fig. 3:**
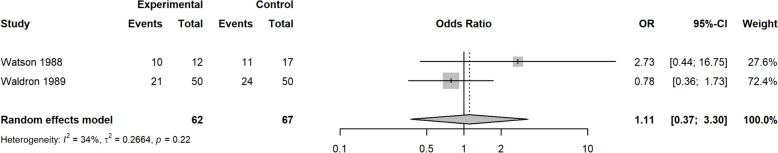
Forest plots of residual septal deformity

**Fig. 4:**
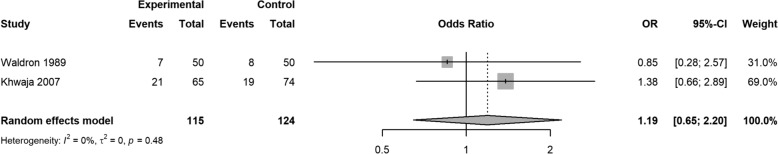
Forest plots of the need for further surgery

**Fig. 5:**
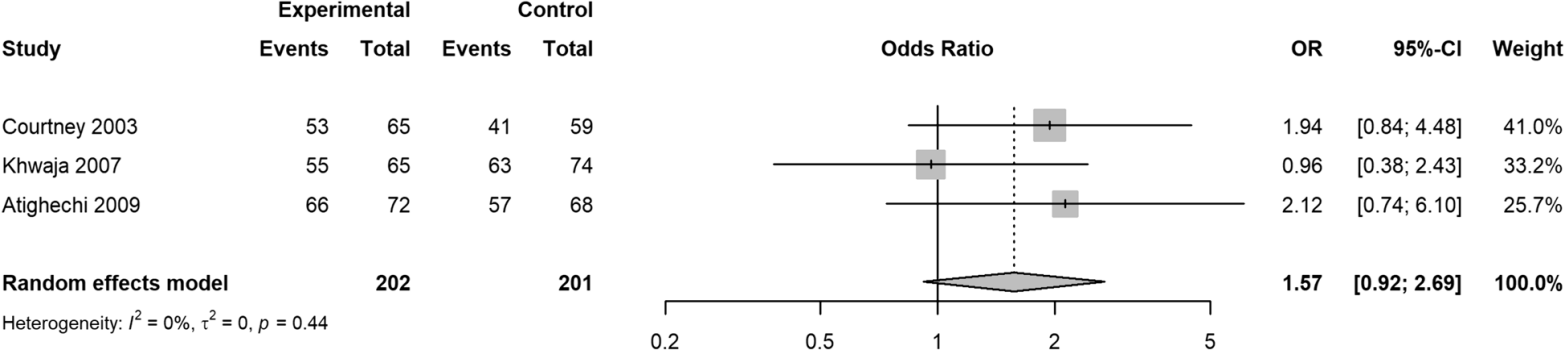
Forest plot of patients’ satisfaction with the anesthetic procedure

**Fig. 6:**
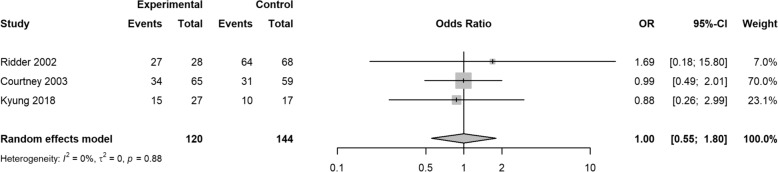
Forest plot of patients’ satisfaction with the surgery results

In this meta-analysis, two studies reported cosmetic results; the pooled OR was 0.70 (95% CI 0.18, 2.64; *z* = − 0.53, *p* = 0.5957) (Fig. 2). Two studies reported results on residual septal deformity; the pooled OR was 1.11 (95% CI 0.37, 3.30; *z* = 0.18, *p* = 0.8558) (Fig. 3). Two studies reported outcomes for the need for further surgery, with pooled OR of 1.19 (95% CI 0.65, 2.20; *z* = 0.56, *p* = 0.5760) (Fig. 4). Three included studies reported patients’ satisfaction with the anesthesia procedure, with pooled OR of 1.57 (95% CI 0.92, 2.69; *z* = 1.65, *p* = 0.0982) (Fig. 5). Three studies reported patients’ satisfaction with the surgery results, with pooled OR of 1.00 (95% CI 0.55, 1.80; *z* = − 0.00, *p* = 0.9974) (Fig. 6).

### Publication *bias*

Deek’s tests for publication bias yielded p values of 0.9634 and 0.5693 for the analyses of patients’ satisfaction with the anesthesia procedure, and patients’ satisfaction with the surgery results, respectively, which revealed that there was no statistically significant publication bias.

### Sensitivity analysis

Sensitivity analysis was not performed because of the limited number of studies included in each pooled analysis.

## Discussion

Nasal bone fractures are the most common injury in the craniofacial region [23]. Current evidence suggests that closed reduction under LA is an acceptable surgery and is not significantly superior to GA in terms of pain, function, and esthetic effects [24]. The primary purpose of this meta-analysis was to compare the effects of GA and LA on the manipulation of NBFs by pooling evidence from published citations.

The results of pooled analyses illustrated that no significant differences were detected between GA and LA in the management of NBFs with regard to the assessment of cosmetic results, residual septal deformity, the need for further surgery, patient satisfaction with the anesthetic procedure, and patient satisfaction with the surgery results. Esthetic deformities with or without airway obstruction are the main indications for NBFs surgeries [8]. The overall deformity and the overall nasal obstruction rates were 10.4% and 10.5% which were the subsequences of NBFs [25]. The findings were similar to those in previous small sample-sized studies which showed that the effect of close nasal bone reduction (CNR) in LA and GA is the same, or in other words, the choice of anesthetic manner has no effect on the reduction of nasal fracture [12, 26, 27]. One of the reasons for the insignificant cosmetic results and incidence of residual septal deformity for GA and LA may be the inevitable defects in the study design of component trials; blinding was not reached in each included study, and subsequently, reporting bias may have been involved. The interpretation of the pooled results should be prudent. More well-designed double-blind RCTs are needed to clarify this hypothesis. Furthermore, it is reported that NBF patients may require further procedures in 9–50% of cases, open nasal septum plasty may ultimately be performed to achieve acceptable esthetic and functional results [1, 28]. The results of this study indicate that the anesthetic method has no significant impact on the rate of patients who require further surgery; however, more factors should be considered to better understand the mechanism of the attribution to a secondary surgery in pretreated NBF patients. In addition, findings of this study based on a larger sample size indicated that LA is considered a good alternative method for closed reduction of NBFs for simple, mild fractures or re-reduction with no associated septal or tip displacement. When planning the treatment of nasal fractures in the context of clinical practice, it is necessary to grade and consider the deviation of the nasal bridge, nasal septum, and nasal tip, a step-by-step surgical plan must be developed based on research results in order to produce successful and economically effective outcomes. Results of this study showed no difference between LA and GA regarding satisfaction with the anesthesia procedure, which suggested favorable and comparable tolerability of both anesthetic methods. Of note, results of Al-Moraissi et al.’s meta-analysis manifested that there was a statistically significant difference between LA and GA for closed reduction of NBFs with regard to patient’s satisfaction with appearance of their nose, which was inconsistent with the current study, the difference may be caused by the heterogeneity of enrolled studies in two meta-analyses [29].

In this meta-analysis, a systematic search of online databases was conducted to identify eligible studies. Two reviewers independently performed study selection and data extraction. Furthermore, heterogeneity between the component studies was appraised. Low-to-moderate heterogeneity was detected among the included studies. Deek’s funnel plot asymmetry tests for publication bias suggested a statistically insignificant publication bias in the meta-analyses of patient satisfaction with the anesthesia procedure and patient satisfaction with the surgery results. Although relevant meta-analysis has been published, controversies across results remain [29, 30]. The results of this updated meta-analysis may provide evidence for a head-to-head comparison between GA and LA in the management of NBFs and clinical hints for practitioners in the choice of anesthetic method in the surgery for NBFs. However, this meta-analysis had some limitations. First, the assessment of patients’ satisfaction with the anesthetic procedure, and patients’ satisfaction with the surgery results were on a subjective basis which may add reporting bias to the pooled outcomes. Second, the number of included studies in each pooled analysis was limited, and more relevant and high-quality studies, especially double-blind RCTs, are warranted in the future to enhance the power of the overall effect sizes. Third, subgroup analysis could not be performed due to limited data extracted and the valid number of enrolled studies in each subgroup; possible impact factors such as the severity of NBFs, dosage of anesthetics on the overall outcomes were not evaluated.

## Conclusions

Based on the outcomes of this study, we may conclude that an insignificant difference was observed between GA and LA in the manipulation of patients with NBFs, and that the choice of anesthetic approach should be based on the tolerability of the methods and severity of nasal fractures.

### Supplementary Information

**Supplementary Material 1.**

## Data Availability

The datasets are available from the corresponding author on reasonable request.
