# Supplementary material for: Comparison of outcomes for general and local anesthesia in the management of nasal bone fractures: a meta-analysis
Source: Eur J Med Res. 2024 Jun 2;29:306. doi: 10.1186/s40001-024-01896-3 (PMC11145784; doi:10.1186/s40001-024-01896-3)
Supplement: Supplementary file 1 — Supplementary Material 1. [file 40001_2024_1896_MOESM1_ESM.docx]

**Table S1** Search strategies for online databases

**PubMed**

| Search number | Query | Results |
| --- | --- | --- |
| 4 | ((((nasal bone fracture[Title/Abstract]) OR (NBFs[Title/Abstract])) OR (nose bone fractures[Title/Abstract])) OR (nasal fractures[Title/Abstract])) AND (((((((((((((((((((((((((general anesthesia[Title/Abstract]) OR (local anesthesia[Title/Abstract])) OR (Anesthesia[Title/Abstract])) OR (Anesthesia[MeSH Terms])) OR (Anesthesia, Cardiac Procedures[Title/Abstract])) OR (Anesthesia, Conduction[Title/Abstract])) OR (Anesthesia, Epidural[Title/Abstract])) OR (Anesthesia, Local[Title/Abstract])) OR (Anesthesia, Spinal[Title/Abstract])) OR (Nerve Block[Title/Abstract])) OR (Anesthesia, Dental[Title/Abstract])) OR (Hypnosis, Dental[Title/Abstract])) OR (Anesthesia, General[Title/Abstract])) OR (Anesthesia, Inhalation[Title/Abstract])) OR (Anesthesia, Rectal[Title/Abstract])) OR (Balanced Anesthesia[Title/Abstract])) OR (Anesthesia, Intravenous[Title/Abstract])) OR (Anesthesia, Obstetrical[Title/Abstract])) OR (Cryoanesthesia[Title/Abstract])) OR (Electroacupuncture[Title/Abstract])) OR (Geriatric Anesthesia[Title/Abstract])) OR (Hypnosis, Anesthetic[Title/Abstract])) OR (Hypnosis, Dental[Title/Abstract])) OR (Neuroanesthesia[Title/Abstract])) OR (Pediatric Anesthesia[Title/Abstract])) | 157 |
| 3 | ((((nasal bone fracture[Title/Abstract]) OR (NBFs[Title/Abstract])) OR (nose bone fractures[Title/Abstract])) OR (nasal fractures[Title/Abstract])) AND (((((((((((((((((((((((((general anesthesia[Title/Abstract]) OR (local anesthesia[Title/Abstract])) OR (Anesthesia[Title/Abstract])) OR (Anesthesia[MeSH Terms])) OR (Anesthesia, Cardiac Procedures[Title/Abstract])) OR (Anesthesia, Conduction[Title/Abstract])) OR (Anesthesia, Epidural[Title/Abstract])) OR (Anesthesia, Local[Title/Abstract])) OR (Anesthesia, Spinal[Title/Abstract])) OR (Nerve Block[Title/Abstract])) OR (Anesthesia, Dental[Title/Abstract])) OR (Hypnosis, Dental[Title/Abstract])) OR (Anesthesia, General[Title/Abstract])) OR (Anesthesia, Inhalation[Title/Abstract])) OR (Anesthesia, Rectal[Title/Abstract])) OR (Balanced Anesthesia[Title/Abstract])) OR (Anesthesia, Intravenous[Title/Abstract])) OR (Anesthesia, Obstetrical[Title/Abstract])) OR (Cryoanesthesia[Title/Abstract])) OR (Electroacupuncture[Title/Abstract])) OR (Geriatric Anesthesia[Title/Abstract])) OR (Hypnosis, Anesthetic[Title/Abstract])) OR (Hypnosis, Dental[Title/Abstract])) OR (Neuroanesthesia[Title/Abstract])) OR (Pediatric Anesthesia[Title/Abstract])) | 175 |
| 2 | ((((((((((((((((((((((((general anesthesia[Title/Abstract]) OR (local anesthesia[Title/Abstract])) OR (Anesthesia[Title/Abstract])) OR (Anesthesia[MeSH Terms])) OR (Anesthesia, Cardiac Procedures[Title/Abstract])) OR (Anesthesia, Conduction[Title/Abstract])) OR (Anesthesia, Epidural[Title/Abstract])) OR (Anesthesia, Local[Title/Abstract])) OR (Anesthesia, Spinal[Title/Abstract])) OR (Nerve Block[Title/Abstract])) OR (Anesthesia, Dental[Title/Abstract])) OR (Hypnosis, Dental[Title/Abstract])) OR (Anesthesia, General[Title/Abstract])) OR (Anesthesia, Inhalation[Title/Abstract])) OR (Anesthesia, Rectal[Title/Abstract])) OR (Balanced Anesthesia[Title/Abstract])) OR (Anesthesia, Intravenous[Title/Abstract])) OR (Anesthesia, Obstetrical[Title/Abstract])) OR (Cryoanesthesia[Title/Abstract])) OR (Electroacupuncture[Title/Abstract])) OR (Geriatric Anesthesia[Title/Abstract])) OR (Hypnosis, Anesthetic[Title/Abstract])) OR (Hypnosis, Dental[Title/Abstract])) OR (Neuroanesthesia[Title/Abstract])) OR (Pediatric Anesthesia[Title/Abstract]) | 313,839 |
| 1 | (((nasal bone fracture[Title/Abstract]) OR (NBFs[Title/Abstract])) OR (nose bone fractures[Title/Abstract])) OR (nasal fractures[Title/Abstract]) | 769 |

**Embase**

| No. | Query | Results |
| --- | --- | --- |
| #4 | #1 AND #2 AND [english]/lim | 128 |
| #3 | #1 AND #2 | 203 |
| #2 | anesthesia:ab,ti OR 'anesthesia'/exp OR 'anaesthesia':ab,ti OR 'anaesthesia, auto':ab,ti OR 'anaesthetic action':ab,ti OR 'anesthesia, auto':ab,ti OR 'anesthetic action':ab,ti OR 'anesthetization':ab,ti OR 'animal anaesthesia':ab,ti OR 'animal anesthesia':ab,ti OR 'autoanaesthesia':ab,ti OR 'autoanesthesia':ab,ti OR 'drop mask anaesthesia':ab,ti OR 'drop mask anesthesia':ab,ti OR 'narcosis':ab,ti OR 'neuroanaesthesia':ab,ti OR 'neuroanesthesia':ab,ti OR 'short anaesthesia':ab,ti OR 'short anesthesia':ab,ti OR 'short duration anaesthesia':ab,ti OR 'short duration anesthesia':ab,ti OR 'anesthesia':ab,ti OR 'general anesthesia':ab,ti OR 'local anesthesia':ab,ti | 542522 |
| #1 | 'nose fracture'/exp OR 'broken nose':ab,ti OR 'external nose fracture':ab,ti OR 'fractura nasi':ab,ti OR 'fracture, nose':ab,ti OR 'fractured nasal bone':ab,ti OR 'fractured nose':ab,ti OR 'nasal bone fracture':ab,ti OR 'nasal fracture':ab,ti OR 'nose bone fracture':ab,ti OR 'nose fracture':ab,ti OR nbfs:ab,ti | 1711 |

**Web of Science**

| # | Search Query | Results |
| --- | --- | --- |
| 1 | nasal bone fracture (Topic) | 935 |
| 2 | general anesthesia (Topic) OR local anesthesia (Topic) | 72201 |
| 3 | #2 AND #1 | 153 |
| 4 | #2 AND #1 and English (Languages) | 118 |
